# Engagement Rules That Underpin DBL-DARC Interactions for Ingress of *Plasmodium knowlesi* and *Plasmodium vivax* Into Human Erythrocytes

**DOI:** 10.3389/fmolb.2018.00078

**Published:** 2018-08-27

**Authors:** Manickam Yogavel, Jyoti Chhibber-Goel, Abhishek Jamwal, Swati Gupta, Amit Sharma

**Affiliations:** Molecular Medicine - Structural Parasitology Group, International Centre for Genetic Engineering and Biotechnology, New Delhi, India

**Keywords:** Duffy antigen receptor for chemokines (DARC), Duffy binding-like domains (DBLs), malaria, *Plasmodium*, Structure

## Abstract

Malaria parasite erythrocytic stages comprise of repeated bursts of parasites via cyclical invasion of host erythrocytes using dedicated receptor-ligand interactions. A family of erythrocyte-binding proteins from *Plasmodium knowlesi* (*Pk*) and *Plasmodium vivax* (*Pv*) attach to human Duffy antigen receptor for chemokines (DARC) via their Duffy binding-like domains (DBLs) for invasion. Here we provide a novel, testable and overarching interaction model that rationalizes even contradictory pieces of evidence that have so far existed in the literature on *Pk*/*Pv*-DBL/DARC binding determinants. We further address the conundrum of how parasite-encoded *Pk*/*Pv*-DBLs recognize human DARC and collate evidence for two distinct DARC integration sites on *Pk*/*Pv*-DBLs.

## Introduction

**Figure 1:**
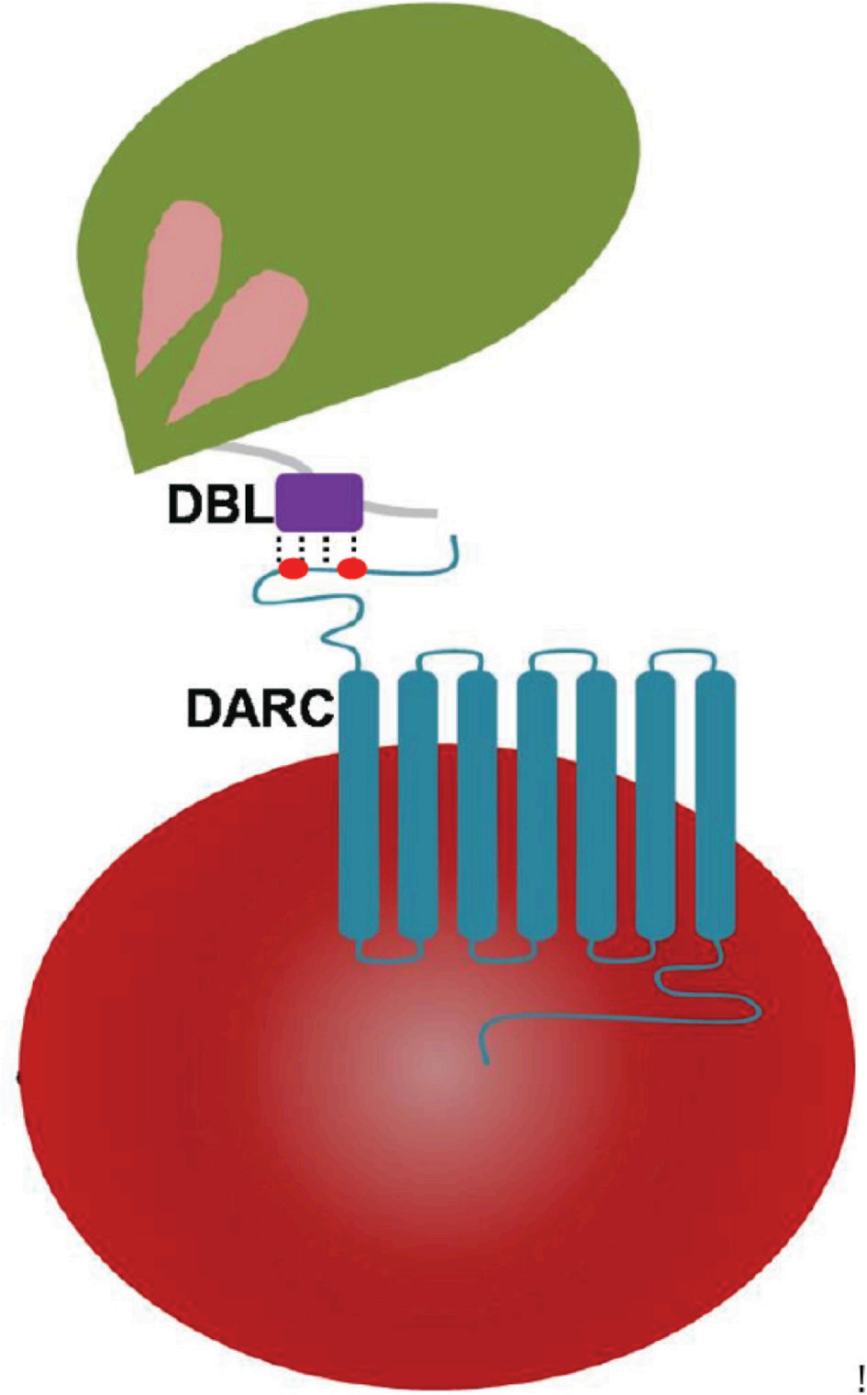
Pictorial representation of *Plasmodium* DBL domain interaction with 7 transmembrane DARC on human erythrocytes. The DBLs are part of *Plasmodium*-encoded membrane proteins called erythrocyte-binding proteins (EBPs) that drive receptor recognition between merozoites (green) and RBCs (red). The DARC extracellular domain of residues 1–60 contains two key tyrosyl residues at positions of 30 and 41 that are post-translationally sulfated—and are marked in red dots.

Engagements of specific host receptors with parasite-encoded ligands between human erythrocytes and malaria parasite surface proteins are key events in invasion of merozoites into erythrocytes (Cowman et al., 2017). The signature Duffy binding-like domain (DBL) is present in parasite-encoded erythrocyte binding proteins (EBPs) and in protein families like *Plasmodium falciparum* (*Pf*) erythrocyte membrane protein 1 (EMP1; Cowman et al., 2017). The latter are part of the *var* gene family where they assist in cytoadherence—these contain several copies of DBLs in each protein, whereas the *Pf*-EBP named EBA-175 harbors two copies (F1 and F2) of DBLs (Mayor et al., 2005; Tolia et al., 2005). In contrast EBPs of *Plasmodium knowlesi* (*Pk*) and *Plasmodium vivax* (*Pv*) contain only single copy of DBL each. There are multiple EBPs present in the malaria parasite *Pk* (α, β, and γ), and it is the *Pk*α-DBL and *Pv*-DBL that mediate Duffy antigen receptor for chemokines (DARC)-dependent invasion of erythrocytes in humans (Choe et al., 2005; Hans et al., 2005; Chitnis and Sharma, 2008). Other DBL variants of *Pk*-EBPs: β and γ likely use alternate receptors on rhesus/human erythrocytes and may mediate invasion by Duffy antigen-independent pathways (Chitnis and Miller, 1994; Cowman et al., 2017). The *Pk/Pv*-DBLs (as part of their EBPs) are present on merozoite surface and are responsible for binding to the DARC receptor on reticulocytes (Figure 1) and thence mediating junction formation that is vital for the parasite invasion process (Adams et al., 1990, 1992; Cowman and Crabb, 2006; Cowman et al., 2017).

The *Pv/Pk*-DBLs specifically recognize DARC's extracellular domain and its sulfated tyrosines via intermolecular interactions (Choe et al., 2005; de Brevern et al., 2005; Chitnis and Sharma, 2008). *Pv/Pk*-DBLs are organized into three sub-domains and are typified (mostly) by 12-cysteine residues that are disulfide linked (Singh et al., 2003, 2006). The importance of *Pv*-DBL/DARC pairing is underscored by human genetic data where DARC negative individuals tend to be protected from *Pv* infection (Michon et al., 2001; King et al., 2011). Contrary to the established DBL-DARC invasion pathway, there is evidence for DARC-independent invasion pathway in case of *Pv* infections, hence calling for new perspectives in assessing the utility of DARC recognizing *Pv*-DBL as a *Pv* vaccine candidate (Ryan et al., 2006; Cavasini et al., 2007; Ménard et al., 2010; Wurtz et al., 2011; Ntumngia et al., 2016).

### Duffy antigen receptor for chemokines (DARC)

**Figure 2:**
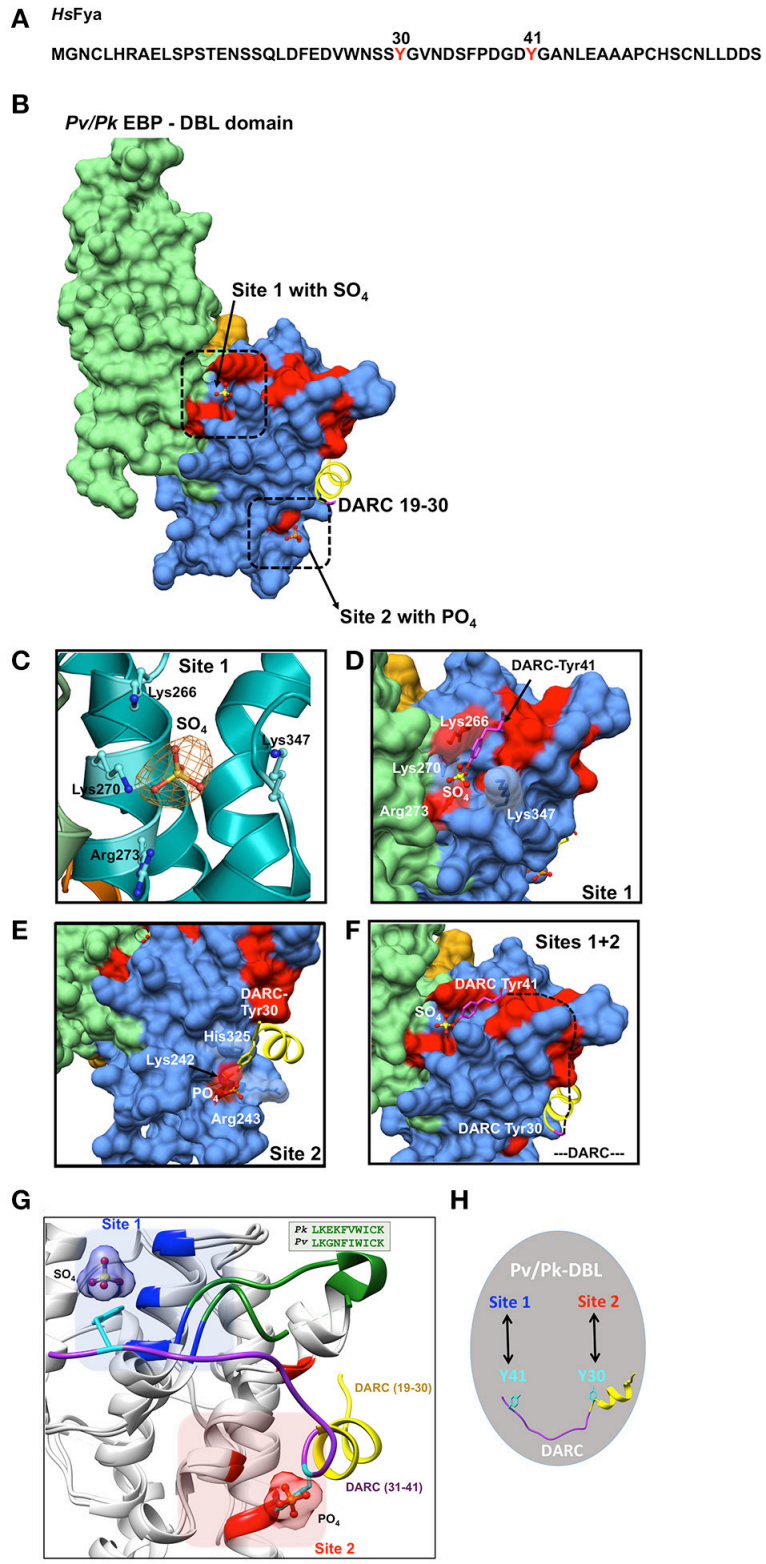
Binding sites on *Pk/Pv*-DBLs for sulfated tyrosines of DARC. **(A)** Sequence of human DARC (*Hs*Fya) with tyrosyl residues 30 and 41 (red) is shown. Tyr30 and Tyr41 are sulfated in human DARC and it has been proposed that sulfation of Tyr 41 is important for high affinity *Pk/Pv*-DBL/DARC engagement (Choe et al., 2005). **(B)** *Pk/Pv*-DBL subdomains 1 (orange), 2 (blue) and 3 (green) are shown as molecular surfaces. The bound sulfate in *Pk*-DBL at Site 1, phosphate in *Pv*-DBL at Site 2, and the N-terminal DARC peptide as ribbon (yellow) for DARC residues 19–30 are shown. Sites 1 and 2 were suggested based on mutagenesis data on *Pv*-DBL (VanBuskirk et al., 2004; Hans et al., 2005). The sulfate at Site 1, phosphate at Site 2 and DARC residues 19–30 at Site 2 are based on crystal structures of *Pv/Pk*-DBLs. The *Pv*-DBL residues whose mutagenesis in two different studies affects the binding of *Pv*-DBL with DARC are marked in red. The yellow region (19–30 of DARC) is from crystal structure of *Pv*-DBL/DARC complex. **(C)** The SA-OMIT (orange) map contoured at 3 σ level for bound SO_4_ in *Pk*-DBL (PDB: 5X6N) at Site 1 is shown. The residues Lys 266, Lys 270, Arg 273, and Lys 347 are identical in *Pv* and *Pk*-DBLs that bind DARC. Note that the corresponding residue numbers are for *Pk*-DBL sequence. **(D)** Zoomed view of Site 1 on *Pv/Pk*-DBLs with sulfate interacting residues (in white) that are identical between *Pk/Pv-*DBLs. The DARC Tyr41 (magenta) is modeled to show its proximity to the sulfate position found in crystal structure of *Pk*-DBL (PDB: 5X6N). The underlying red patches of *Pv*-DBL residues mark the path taken by those whose mutagenesis in two different studies effects binding of *Pv*-DBL with DARC (VanBuskirk et al., 2004; Hans et al., 2005). **(E)** Zoomed view of Site 2 on *Pv*-DBL modeled after superposition of two *Pv*-DBL structures (PDB IDs: 3RRC and 4NUV) with bound phosphate and unsulfated DARC peptide (residues 19–30) is shown. Note that the corresponding residue numbers are for *Pv*-DBL (PDB: 3RRC). The underlying red patches of *Pv*-DBL residues mark the path taken by the residues whose mutagenesis in two different studies effects binding of *Pv*-DBL with DARC (VanBuskirk et al., 2004; Hans et al., 2005). **(F)** Modeling of *Pv*-DBL with DARC region from 19 to 41 where DARC peptide (19–30, in yellow) and the bound sulfate (from *Pk*-DBL, in yellow) are shown. The dashed black line (DARC residues 31–40) shows the proposed trajectory of DARC peptide once its Tyr30 is bound to Site 2 and its Tyr41 is bound to Site 1. Again, note that many residues that are critical (colored red) for DARC binding, as revealed by two separate mutagenesis studies, fall along the path from Site 2 to Site 1 resulting in a wide DARC binding footprint (VanBuskirk et al., 2004; Hans et al., 2005). **(G)** Structural superimposition of *Pk*-DBL (5X6N) and *Pv*-DBL (4NUV) that bind DARC are shown in gray ribbons. Sulfate at Site 1 (blue) and Tyr41 (cyan) are shown along with their interacting residues (blue) from *Pk*-DBL (5X6N). Phosphate at Site 2 (red) and Tyr30 (cyan) are shown along with their interacting residues (red) from *Pv*-DBL. The loop between α6 and α7 of *Pv/Pv*-DBLs spanning residues 338–347 (*Pk*-DBL) and 369–378 (*Pv*-DBL) are in green. The DARC peptide spanning residues 19–41 (19–30 in yellow and 31–41 in purple) are docked on to *Pk/Pv*-DBLs such that it interacts via Tyr41 on Site 1 and via Tyr 30 on Site 2. **(H)** Proposed model on the modes of interaction between *Pv/Pk*-DBLs and sulfated DARC. It is proposed that soluble region of DARC peptide (from residues 19 to 41), via its sulfated Tyr41 and Tyr30 (cyan), engages at site 1 and 2 respectively on *Pv/Pk*-DBLs. This testable model is a dual-site mode of DARC's engagement with *Pv/Pk*-DBLs and can be experimentally assessed.

DARC is a seven-transmembrane protein present on surface of erythrocytes and endothelial cells (de Brevern et al., 2005). It is a promiscuous cytokine/chemokine receptor involved in pro-inflammatory processes of the immune system (Rot and von Andrian, 2004; Cowman et al., 2017). DARC is also used as an entry vehicle by malaria parasites *Pk* and *Pv* (Miller et al., 1975, 1976; Chitnis and Sharma, 2008). The binding requirements on DARC for (*Pv*)-DBL interaction have been previously described (Choe et al., 2005; Tournamille et al., 2005). Critical (*Pv*)-DBL binding residues have been mapped to an N-terminal region of DARC (Tournamille et al., 2005). Latter contains two key tyrosine residues at positions 30 and 41 whose post-translational modifications in the form of sulfations have been reported (Choe et al., 2005; Figure 2A). Sulfated Tyr41 has been considered a dominant motif and essential for (*Pv/Pk*)-DBL/DARC binding (Choe et al., 2005). As per report sulfated Tyr30 did not affect direct binding of (*Pv*)-DBL with DARC, but contributed to IL8 association with DARC (Choe et al., 2005).

### Binding sites on *Pk/Pv*-DBLs for sulfated tyrosines of DARC

The *Pk/Pv*-DBLs and their three subdomains contain numerous disulfide linked cysteine residues that are largely conserved, and these likely contribute to DBLs' structural integrity (Singh et al., 2003, 2006; Gill et al., 2009). Conservation of hydrophobic residues and variance in solvent exposed residues within DBLs possibly allows for a parasite-specific, evolutionarily useful structural motif that can be both constant (in structural terms) and variable (in sequence). Hence, DBLs' seem to have evolved using the same principles as antibody structures, where the overall 3D core structures remain similar but sequence variation in exposed residues and loop regions allows for surface diversity that can thus engage with plethora of bio-molecular receptors.

In efforts to understand the structural underpinnings of *Pk/Pv*-DBL/DARC complex, the resolved crystal structure of *Pk*-DBL had suggested a region on its subdomain 2 that could accommodate the DARC's sulfated tyrosine (Singh et al., 2006), and serve as its recognition site (here referred to as Site 1, Figures 2B,C). The *Pk*-DBL subdomain 2 presents a remarkably surface exposed region of highly conserved residues that arrange into distinct regions lying adjacent to each other—formed by positively charged residues (Lys96, Lys100, Arg103, and Lys177) and non-polars (Tyr94, Leu168, and Ile175; Singh et al., 2006). These two dual-charge/hydrophobicity character surfaces on *Pk*-DBL were proposed to engage with DARC's sulfated tyrosyl based on structural considerations, sequence conservation patterns, atomic properties, and experimental data emanating from collation of mutagenesis experiments from two distinct groups at the time (VanBuskirk et al., 2004; Hans et al., 2005). Further, the proposed residues on *Pk*-DBL were invariant in the DARC binding *Pv*-DBL, hence lending support to the proposal of their essential role in acting as a recognition site for DARC's sulfated tyrosine (VanBuskirk et al., 2004; Choe et al., 2005; Hans et al., 2005; Singh et al., 2006). These data, coupled with the observation that the sulfated Tyr41 of DARC allows strong binding to *Pk/Pv*-DBLs (Choe et al., 2005) led to a model of DARC's docking onto *Pk/Pv*-DBLs via the identified site (here labeled as Site 1, Figures 2C,D). The crystal structures of *Pv*-DBLs (PDBs: 3RRC, 4NUU, and 4NUV) later identified another site for DARC binding, hereafter labeled Site 2 (Figure 2B), where based on (a) the binding of crystallization liquor phosphates (possibly mimicking the sulfate of DARC's sulfated Tyr30), and (b) DARC peptide spanning residues 19–30, a key site for DARC binding was proposed (Batchelor et al., 2011, 2014). In these above studies, it is to be noted that the *Pk/Pv*-DBLs were bacterially produced, and the DARC peptide used for co-crystallization was evidently not sulfated (see PDBs 4NUU and 4NUV). The *Pv*-DBL/DARC engagement was further proposed to lead to dimerization of *Pv*-DBL (Batchelor et al., 2011, 2014). The existence of this DARC binding site (Site 2 in this review) was proposed based on the evidence of bound phosphate (PDB: 3RRC) at the (proposed) dimer interface of *Pv*-DBL (Batchelor et al., 2011), and then in a latter study of *Pv*-DBL/DARC complex, several DARC residues (numbers 19–30) were found in proximity to Site 2 (Figures 2B,E)—although the bound Tyr30 was not sulfated (Batchelor et al., 2011, 2014). The above sets of structural and biochemical data led to a conundrum on the location of the binding site for DARC's sulfated Tyr41 on *Pk/Pv*-DBLs, and its significance in light of a previous report (Choe et al., 2005) that had suggested that sulphation of Tyr41 was an essential factor for coupling of DARC with *Pk/Pv*-DBLs (Choe et al., 2005). We shall return to this in a later section.

It has been also proposed that *Pv*-DBL engagement with DARC extracellular domain drives dimerization of *Pv*-DBLs (PDB IDs: 3RRC, 4NUU, and 4NUV; Batchelor et al., 2011, 2014). Extensive analysis using PISA (http://www.ebi.ac.uk/pdbe/pisa/) and EPPIC (http://www.eppic-web.org/), two software that analyze the oligomeric states of biomolecular structures deposited in the public database PDB (Krissinel and Henrick, 2007; Duarte et al., 2012; Baskaran et al., 2014), does not strongly support dimerization of *Pv*-DBL when bound to phosphate or to the DARC peptide spanning residues 19–30 (we encourage readers to assess this using online PISA and EPPIC software and the PDBs: 3RRC, 4NUU, and 4NUV). However, we do not preclude the oligomerization of *Pv-*DBLs when bound to DARC *in vivo*, and/or in the context of full-length proteins of each when they interact on their respective cell surfaces. Yet from the available structural data in PDB, it can be construed that the *Pv/Pk*-DBL monomer contains most vital structural elements required for engagement with two sites on sulfated DARC. Indications of dimeric states gathered from *in solution* studies like SAXS, ITC and analytical ultracentrifugation do not necessarily reveal the exact binding interfaces in a biomolecular complex. The structural resolution of *Pk/Pv-*EBPs bound to fully sulfated DARC will eventually reveal the modes of complex formation between these receptor ligand pairs.

### A new overarching model for coupling of *Pk/Pv*-DBLs with DARC

The *Pv-*DBL (PDB: 3RRC) has two bound phosphates near/at Site 2, where later DARC peptide residues (19–30) were also found (PDBs: 4NUU, 4NUV). During process of routine structure re-refinement of deposited crystal structures, as recommended (Read et al., 2011) due to the more robust convergence routines and higher quality of resulting stereochemical models for proteins, we observed a bound sulfate at Site 1 in the *Pk*-DBL crystal (PDB: 5X6N, Figures 2B,C). The bound sulfate site overlaps with the earlier proposed sulfa-tyrosine recognition region (Singh et al., 2006), nestled by highly conserved positively charged residues—most of which were earlier mapped based on mutagenesis screens (VanBuskirk et al., 2004; Choe et al., 2005; Hans et al., 2005; Singh et al., 2006). Indeed, both *Pv/Pk*-DBLs have other phosphate/sulfate binding sites in their crystal structures but we have taken into account only those that are proximal to the regions where mutagenesis and biochemical data together provide strong evidence for DARC engagement (VanBuskirk et al., 2004; Hans et al., 2005; Sampath et al., 2013). The *Pk*-DBL protein migrates as a monomer on gel filtration column in the absence of detergent, and in the crystal structure (PDB: 5X6N) it is also an unambiguous monomer—even when bound to a SO_4_ in Site 1 (PDB ID: 5X6N and kindly assess via PISA/EPPIC). The likely irrelevant lone detergent binding site in *Pk*-DBL (PDB: 5X6N) is distal from both Sites 1 and 2. The presence of bound sulfate at Site 1 in *Pk*-DBL is clearly a striking observation, and it immediately opens the possibility of reassessing and reinterpreting the engagement rules for DARC recognition and binding by *Pk/Pv*-DBLs.

Therefore, based on (a) *Pk*-DBL-sulfate complex (Site 1, Figures 2B–D), (b) *Pv*-DBL-phosphate complex (Site 2, Figures 2B,E), (c) *Pv*-DBL-DARC peptide complex (Site 2, Figures 2B,E) and available mutagenesis and biochemical data on *Pv*-DBL/DARC interactions (VanBuskirk et al., 2004; Hans et al., 2005; Sampath et al., 2013), we are able to propose a simple, novel, testable and largely rationalized resolution to the conundrum of *Pk/Pv*-DBL/DARC coupling (Figures 2F,G). We envisage that the DARC peptide 1–60 may dock on to *Pk/Pv*-DBLs via its sulfated Tyr41 on Site 1 (Figures 2B,F,H; PDB: 5X6N) and on to Site 2 via the DARC peptide containing residues 19 to 30 (Figures 2F,G; PDB 4NUV). Structural, molecular size, and stereochemical considerations will allow the DARC peptide (spanning residues 19–41) to traverse from Site 2 (where Tyr 30 is) to Site 1 (where Tyr 41 is) on *Pk/Pv*-DBLs. Site 1 on *Pk/Pv*-DBLs is located on N-cap region of α2 helix and the loop between α6 and α7 helices, while Site 2 is rests on α1, α5, and α6 helices (Figure 2G). The loop (residues: 338–347 in *Pk*-DBL, 369–378 in *Pv*-DBL) between α6 and α7 is highly conserved in *Pk/Pv*-DBLs (Figure 2G). It is noteworthy that this loop is partly disordered in *Pv*-DBL (4NUV), but is ordered in sulfate bound crystal structure of *Pk*-DBL (5X6N).

A dual-site DARC hooking on to *Pk/Pv*-DBLs offers a more complete binding footprint of DARC (Figure 2G). Indeed, the molecular size of a sulfated tyrosine (from its C-α to sulfate) is ~14Å (Figure 2C). Structural modeling of the atomic space between *Pk*-DBL's Tyr94 (Site 1 residue, i.e., Tyr 264, based on full length *Pk*-DBL, as in Singh et al., 2006) and the bound sulfate shows that a sulfated tyrosine will fit snugly into it (Figure 2C). Further, the side chains in *Pk/Pv*-DBLs that constitute Site 1 are identical (4/4), or mostly (2/3) conserved for Site 2 (Figures 2D,E). Invariance in Site 1 residues of *Pk/Pv*-DBLs: Lys96, Lys100, Arg103, and Lys 177 (*Pk*-DBL numbering, as in Singh et al., 2006) that nestle (via charge complementarity) the bound sulfate moiety (Figures 2C,D), and its potential as a sulfa-tyrosine recognition site based on earlier mutagenesis experiments together provide very strong support for the significance of Site 1 in *Pk/Pv*-DBLs/DARC interactions. The above analyses hence propose that both Sites 1 and 2 are part of the overall binding footprint of DARC on *Pv/Pk*-DBLs, and that Sites 1 and 2 likely represent the regions of engagement where sulfated Tyr41 and Tyr30 (respectively) are recognized by the *Pv/Pk*-DBLs (Figures 2F,G). The intervening regions of *Pv/Pk*-DBL and DARC will also likely make contact with one another, and therefore the mutagenesis footprint on DBLs will be larger than that alone for Sites 1 and 2—as indeed seems to be the case, and shall be discussed below (VanBuskirk et al., 2004; Hans et al., 2005).

Earlier, single amino acid mutagenesis data had suggested over ~two dozen mutations that could effect binding of DARC to *Pv-*DBL (VanBuskirk et al., 2004; Hans et al., 2005). Based on comprehensive collation of structural and experimental data, we now propose a model that shows that majority of the 25 mutations (VanBuskirk et al., 2004; Hans et al., 2005) that diminish *Pv*-DBL/DARC engagement fall in a predicted trajectory that DARC peptide may take when traversing from Site 1 from Site 2 (Figures 2F,G). Two independent mutagenesis screens had earlier identified several sets of residues on *Pv*-DBL that altered binding to DARC—several of these residues line and nestle the sulfate in *Pk*-DBL at Site 1, besides being proximal to Site 2 (VanBuskirk et al., 2004; Hans et al., 2005). Further support for our model comes from a glycan masking study that was focused on *Pv*-DBL/DARC interactions wherein it was suggested that addition of an N-glycan site near the proposed Site 1 (our proposed model), but not in other regions of *Pv*-DBL, including the proposed dimer interface site (Site 2), abrogated DARC binding (Sampath et al., 2013). These data therefore additionally support our envisaged model of twin engagement of DARC via its sulfated Tyr41 at Site 1 and with DARC residues 19–30 at Site 2. Again, a comprehensive mapping of mutagenesis data support the idea that DARC residues 19–41 (via Tyr 30 and Tyr 41) traverse from Site 2 toward Site 1 along the path where numerous mutants map (Figures 2F,G; VanBuskirk et al., 2004; Hans et al., 2005).

It needs to be emphasized again that our proposed model is not discounting the importance of DARC (residues 19–30) identified earlier (Tournamille et al., 2005; Batchelor et al., 2014). Indeed, our new suggested structural framework model of *Pk/Pv*-DBL/DARC interactions supports, collates and integrates many previous studies (VanBuskirk et al., 2004; Choe et al., 2005; Hans et al., 2005; Singh et al., 2006; Batchelor et al., 2011, 2014). Our model is consistent with most mutagenesis and crystallography work published so far, and provides a more comprehensive explanation for DARC's binding via its sulfated Tyr30 and Tyr41 to two binding sites (Sites 1 and 2) each on *Pk*/*Pv*-DBLs (Figures 2G,H). The proposed architecture of the complex sheds new light on DBL-DARC interactions, and our overview suggests clear avenues for experimental validation of the proposal. Our model also offers scope to make new sets of site-directed mutants in *Pk*/*Pv*-DBLs that can directly address the above hypotheses using the available 3D structures.

Finally, the indicated structural framework model of *Pk/Pv*-DBL-DARC significantly resolves the puzzle of recognition site(s) that underpin this complex. Our analyses integrate new evidence for two distinct DARC binding sites on *Pk*/*Pv-*DBLs (called Sites 1 and 2, in the chronological order they were described in the literature) that together likely engage the extracellular domain of DARC via sulfated Tyr41 and Tyr30, respectively (Figures 2G,H). Our analyses shall be important for critical assessment of both malaria vaccine and inhibitor development efforts that are targeted at abrogating *Pk/*Pv-DBL-DARC interactions as a possible avenue to prevent invasion of *Pk/Pv* malaria parasites into human erythrocytes.

## Author contributions

MY, JC-G, AJ, and SG analyzed the data and prepared figures. MY, JC-G, and AS wrote and finalized the manuscript. MY and AS designed the study.

### Conflict of interest statement

The authors declare that the research was conducted in the absence of any commercial or financial relationships that could be construed as a potential conflict of interest.
